# Dual-Band Plasmonic Perfect Absorber Based on Graphene Metamaterials for Refractive Index Sensing Application

**DOI:** 10.3390/mi10070443

**Published:** 2019-07-02

**Authors:** Zao Yi, Cuiping Liang, Xifang Chen, Zigang Zhou, Yongjian Tang, Xin Ye, Yougen Yi, Junqiao Wang, Pinghui Wu

**Affiliations:** 1 Joint Laboratory for Extreme Conditions Matter Properties, Southwest University of Science and Technology, Mianyang 621010, China; 2 Sichuan Civil-Military Integration Institute, Mianyang 621010, China; 3 Research Center of Laser Fusion, China Academy of Engineering Physics, Mianyang 621010, China; 4 College of Physics and Electronics, Central South University, Changsha 410083, China; 5 School of Physics and Engineering and Key Laboratory of Materials Physics of Ministry of Education of China, Zhengzhou University, Zhengzhou 450001, China; 6 Photonic Technology Research & Development Center, Key Laboratory of Information Functional Material for Fujian Higher Education, Quanzhou Normal University, Quanzhou 362000, China

**Keywords:** surface plasmon resonance, graphene, refractive index sensor, metamaterials

## Abstract

We demonstrate a dual-band plasmonic perfect absorber (PA) based on graphene metamaterials. Two absorption peaks (22.5 μm and 74.5 μm) with the maximal absorption of 99.4% and 99.9% have been achieved, respectively. We utilize this perfect absorber as a plasmonic sensor for refractive index (RI) sensing. It has the figure of merit (FOM) of 10.8 and 3.2, and sensitivities of about 5.6 and 17.2 μm/RIU, respectively. Hence, the designed dual-band PA-based RI sensor exhibits good sensing performance in the infrared regime, which offers great potential applications in various biomedical, tunable spectral detecting, environmental monitoring and medical diagnostics.

## 1. Introduction

Surface plasmon resonance (SPR) is the interaction between electromagnetic fields and free electrons in metals [1]. With the development in nano-fabrication technology, SPR has been widely used in the field of optical applications, biological analysis, photocatalysis, and chemical detection [2,3,4,5,6,7,8]. Due to SPR being extremely sensitive to a small change in the refractive index of surrounding medium, many works concentrate on the application of sensing technology [9,10]. As a two-dimensional material with only one atom thickness packed in a honeycomb lattice, graphene has multitude of exceptional electronic and optical properties because of its unique electronic band structures [11,12,13,14,15]. Graphene can excite surface plasmons like metals, but unlike metals the surface plasmons of graphene are tunable. Therefore, graphene can replace metal as a plasmonic sensor. It is common to use the perfect absorption structure of plasmon for sensing. The perfect absorber is a subject worth researching. Perfect absorption can be realized by optimizing the shape, size, and optical properties of the metamaterials of the perfect absorber (PA) [16,17,18,19]. So far, there are many studies on perfect absorbers, including simulations and experiments. For example, in 2011, Zhang et al. designed and manufactured a near-infrared dual-band plasma absorber [20]. In addition, in 2019, Xu et al. proposed and fabricated a metamaterial nearly perfect absorber in the visible and near-infrared region [21]. These reported experimental measurements match well with the simulations. Once the metal structure is fabricated, the resonant wavelength and operating range will be unchangeable. Since graphene has many advantages over metals, we chose it as the research material. At the same time, the perfect absorber with graphene metamaterial can have good refractive index sensing performance. When the refractive index of the environment changes, the perfect absorption is destroyed, thus realizing sensing.

However, at present, many works only operate an a single wavelength, which greatly hinders their application in practice. In many applications, when multiple absorption bands can be monitored, it is convenient to correlate and study structural changes between different molecular regions and is critical to the accurate identification of molecular species [22,23,24,25]. Therefore, from the viewpoint of application, a PA structure with dual or multiple bands is highly advantageous for many occasions.

To this end, we theoretically design a dual-band plasmonic perfect absorber (PA) based on graphene metamaterials in the infrared regime. We obtain two absorption peaks located at *λ*_1_ = 22.5 μm and *λ*_2_ = 74.5 μm with high absorption coefficients of 99.9% and 99.4% respectively. The spectral location of the absorption values can be adjusted actively by tuning the graphene’s Fermi level without changing the geometric parameters of the structure. Besides, the resonant wavelengths of two modes are very sensitive to changes of environmental refractive index. It has the figure of merit (FOM) of 10.8 and 3.2, and sensitivities of about 5.6 and 17.2 μm/RIU (refractive index unit) respectively. The PA-based RI sensor has narrower FWHM (FWHM is the full width of half maximum) than an ordinary absorption sensor, which means the RI sensor has better sensing performance. Hence, the proposed PA-based RI sensor can offer great potential applications in biomedicine, environmental monitoring and medical diagnostics.

## 2. Structure Design and Numerical Model

The finite-difference time-domain (FDTD) method is employed to analyze the optical response in the graphene structures. TM-polarization indicates that the direction of the incident electric field is along the *x* axis. In the FDTD simulation calculations, the accuracy of mesh in the graphene layer along the *x* (*y*) and *z* axes are set to 25 and 0.2 nm. The anti-symmetric and symmetric boundary conditions were respectively adopted in the *x* and *y* directions. The boundary conditions of the perfectly matched layer are used in the *z* direction along the propagation of the incident plane wave.

**Figure 1:**
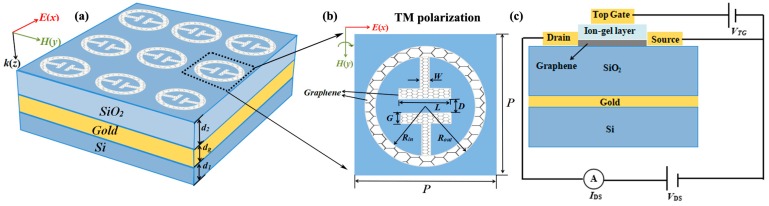
(**a**) Schematic of the perfect absorber structure; (**b**) the incident light polarization configuration (TM-polarization, TM-polarization indicates that the direction of the incident electric field is along the x axis); and (**c**) the schematic geometry of a top gate configuration to manipulate the Fermi energy of graphene. Parameters: *d*_1_ = 3 μm, *d*_g_ = 0.45 μm, *d*_2_ = 4.17 μm, *t* = 1 nm, *R*_in_ = 0.8 μm, *R*_out_ = 1.05 μm, *D* = 0.2 μm, *L* = 0.7 μm, *G* = 0.25 μm, and *W* = 0.25 μm. The periods in both *x* and *y*-directions are 2.5 μm. The whole structure resides on a substrate (*n*_1_ = 3.4).

The geometry of the PA-based RI sensor is depicted in Figure 1. From bottom to top, there is a Si (*n*_1_ = 3.4) layer, a gold mirror, a SiO_2_ (*n*_2_ = 1.97) layer and a periodical graphene pattern tightly stacked to form this structure. Since this structure excites the local surface plasmons, the graphene surface plasmon is confined to the graphene surface—that is to say it does not pass through the SiO_2_ or even reach the gold surface. What really reaches the gold surface should be part of the incident light. One part of the light in the incident light interacts with the graphene. The other part passes through the graphene to reach the gold surface, which is reflected back by the gold and then lost in the SiO_2_. We chose the lowermost layer as the most common material, Si [26]. Its function can be understood as a substrate or a buffer layer to protect the whole device against accidental mechanical damage.

The geometric structure we designed is simple and easy to manufacture. At the same time, considering manufacturing tolerances, we should allow the gap between unit cells to be greater than 100 nm. In each unit cell, the top graphene-pattern composes of a graphene ring and four rectangular graphene bands. *R*_in_ and *R*_out_ are the radius of the inner and outer rings of graphene, respectively. *d*_1_, *d_g_*, *d*_2_, and *t* represent the thickness of Si, gold mirror, SiO_2_ and graphene, respectively. In our calculation, the geometrical parameters are set as *d*_1_ = 3 μm, *d*_g_ = 0.45 μm, *d*_2_ = 4.17 μm, *t* = 1 nm, *R*_in_ = 0.8 μm, *R*_out_ = 1.05 μm, *D* = 0.2 μm, *L* = 0.7 μm, *G* = 0.25 μm, and *W* = 0.25 μm. The periods in both *x* and *y* directions are 2.5 μm. In the whole absorber structure, interspacing of unit elements is 0.4 μm. The total size of each manufactured device is 100 μm.

The designed graphene metamaterials may be realized experimentally by the following procedures [27]: the SiO_2_ spacer and Si substrate are respectively coated on the upper and lower surfaces of Au through thermal evaporation, and then the monolayer graphene is coated on the top of the SiO_2_ spacer after a chemical vapor deposition (CVD), finally the cavity structures are fabricated on the monolayer graphene by electron beam etching.

The complex dielectric constant of gold at in the infrared regime is described by the Drude model with the plasma frequency *w_p_* = 1.37 × 10^16^ rad/s, *e*_¥_ = 1, and the damping constant *g_c_* = 1.224 × 10^14^ rad/s [28,29,30,31].

(1) $$\varepsilon_{Au}\left(\omega \right)=\varepsilon_{\infty }-\omega_{p}{}^{2}/(\omega^{2}+i\gamma_{c}\omega )$$

where *w* is the angular frequency of the incident electromagnetic wave. The surface conductivity of signal-layer graphene *s* can be described by the Kubo formula including interband and intraband transitions [32]. *k*_B_, *T* and *E*_F_ are the Boltzmann constant, temperature, and Fermi level, respectively. When *E*_F_ >> *k*_B_*T*, the interband transition dominates and surface conductivity can be simplified as [33,34,35]:

(2) $$\sigma (\omega )=\frac{e^{2}E_{F}}{\pi \hbar^{2}}\frac{i}{\omega +i/\tau }$$

where *e*, ℏ and *t* represent the charge of an electron, reduced Planck’s constant, and the relaxation of time, respectively. The relaxation time *t* can be expressed as [36,37]:

(3) $$\tau =\frac{\mu E_{F}}{ev_{F}^{2}}$$

where *τ* is dependent on the electron mobility *μ* = 1 × 10^4^ cm^−2^ V^−1^ s^−1^, and the Fermi velocity *ν_F_ =* 1 × 10^6^ ms^−1^. In this work, Fermi level (*E*_F_ = 1.0 eV), and the relaxation of time (*τ =* 1.0 ps) are assumed. Under these conditions, the absorption response can be calculated by using the relation *A* = 1 − *R* [38]. When the *R* (reflection) approaches zero, *A* can obtain perfect absorption.

As predicted from Equation (2), the surface conductivity of graphene can be tuned via manipulating its Fermi energy. At present, low top gate voltage can be used to increase the Fermi energy of graphene. Many gate dielectric materials such as 2D electron gas [39] indium tin oxide [40] monolayer MoS_2_ [41] and ion-gel [42] have been researched in the midinfrared and THz regime. Because ion-gel has good mechanical flexibility, fatigue stability, and excellent electrochemical and thermal stability, it can be compatible with tunable graphene plasmonic devices on various substrates [43,44]. Therefore, the most appropriate and common dielectric is an ion-gel with high capacitance [45]. The schematic geometry of a top gate configuration to control the Fermi energy of graphene is shown in Figure 1c. The ionic gel-layer applied between the graphene and gold electrodes induces carrier concentration and allows the absorber to enter the terahertz band [46,47,48].

## 3. Simulation Results and Discussion

**Figure 2:**
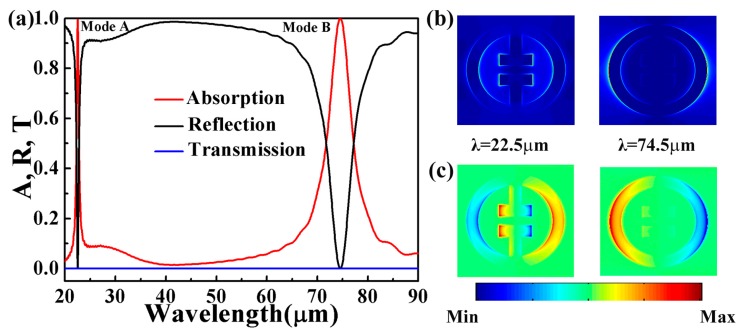
(**a**) Absorption (A), reflective (R), and transmission (T) spectrum of the presented structure; (**b**,**c**) are the electric field distribution of the corresponding graphene and the simulated surface charge density distributions at λ_1_ = 22.5 μm and λ_2_ = 74.5 μm, respectively.

For the whole structure, when parameters are set as mentioned above, two resonance responses with reflection and absorption are shown at around 22.5 μm and 74.5 μm in Figure 2a. The absorption of Mode A and Mode B are 99.4% and 99.9%, respectively. Figure 2b shows the electric field distributions of the corresponding graphene at the resonance. It is observed that the electric field is concentrated mainly at the outer ring, inner edges, and corners of the graphene structure. Figure 2c shows the simulated surface charge density distributions at λ_1_ = 22.5 μm and λ_2_ = 74.5 μm, respectively. For Mode A (λ_1_ = 22.5 μm), the physical origin is related to the quadrupole resonances. For Mode B (λ_2_ = 74.5 μm), the opposite charges are concentrated on both sides of the ring (left and right sides), which indicates the excitation of dipole resonance in the graphene metamaterials array.

**Figure 3:**
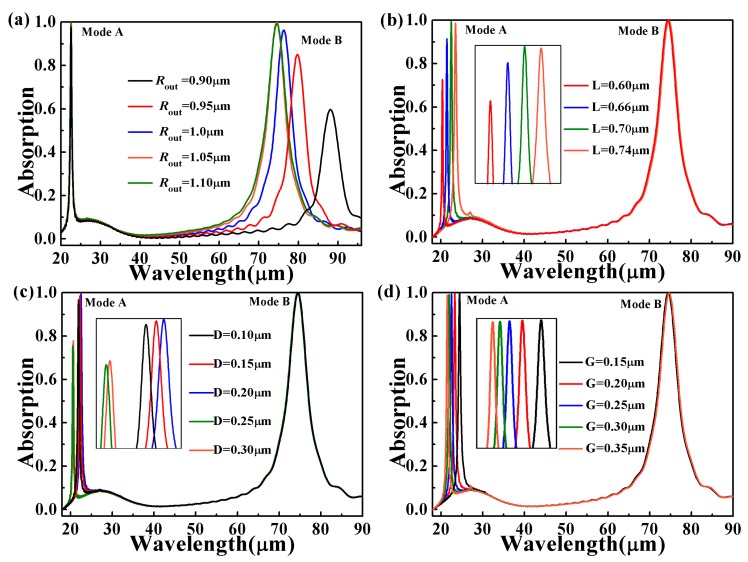
The effect of different parameters of the absorber on the absorption spectrum: (**a**) the outer ring radius of the graphene (*R*_out_); (**b**) *L*, (**c**) *D*, (**d**) *G*. The Fermi level of graphene is 1.0 eV in (b), (c), and (d). The insert graph is the absorption peak of Mode A or Mode B.

To further analyze the performance of dual-band absorber, we first study the effect of different geometric parameters by numerical simulation. Figure 3a shows the absorption spectrum of the dual-band absorber with changing the outer ring radius (*R*_out_) of graphene from 0.8 μm to 1.05 μm. For Mode A, the absorption peak hardly changes. For Mode B, the maximum absorption decreases and peak wavelength experiences a blue shift as the *R*_out_ increases. In Figure 3b, when *L* increases, the distance of graphene arrays in the *x-*direction decreases, resulting in an enhancement in the coupling between them. This is the reason why Mode A experiences red shift and absorption enhancement of when *L* increases. In Figure 3c, for Mode A, different *D* values also affect the absorption and peak wavelength. It can also be seen from Figure 2b that the entire internal structure of the ring contributes to the excitation of mode A. Therefore, changing only one of the internal parameters may cause a complex change in its optical response. So we can see that when *D* increases from 0.1 to 0.2 μm, the Mode A has experienced a red shift, and the absorption at *D* = 0.2 μm is maximum. When *D* increases to 0.25 μm, the positional shift of the peaks is no longer monotonous. This is because when *D* > 0.25 μm, the spacing between the internal structure and the inner ring is reduced, resulting in coupling enhancement and red shift.

When *D* = 0.2 μm, the absorption reaches the maximum. In Figure 3d, as *G* increases, the absorption of Mode B hardly changes, while the absorption of Mode A slightly decreases and peak wavelength experiences a blue shift. In other words, the change of *G* only affects the resonant wavelength of Mode A.

**Figure 4:**
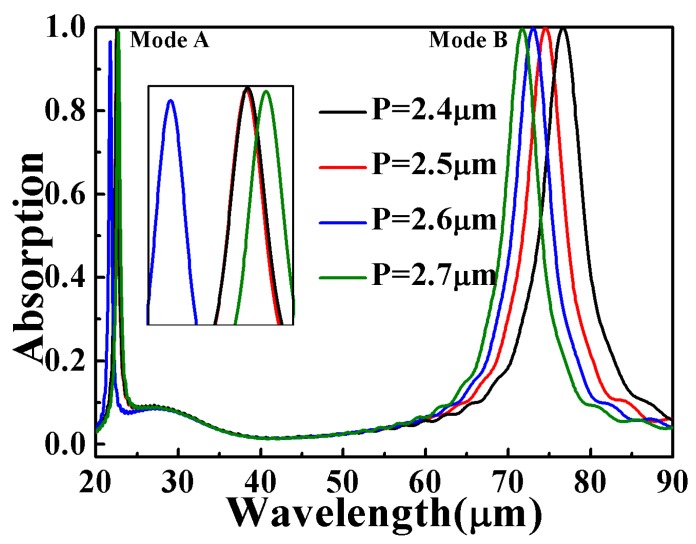
The absorption spectra of the proposed structure with different period *P*. The insert graph is the absorption peak of Mode A.

Figure 4 shows the absorption spectrum with the size of *P* changed from 2.4 to 2.7 μm. For Mode A, the peak first experiences a blue shift and then undergoes a red shift. The maximum absorption can be achieved when *P* is equal to 2.4 or 2.5 μm. For Mode B, as the period varies, there is almost no change in absorption, but the absorption peak wavelength has a significant blue shift with the period increases. This is because the grating period is the determinant factor for the resonant frequency when the grating period *P* matches the period of the plasmonic wave [49]. The change of absorption peak wavelength depends directly on the period of PA, which is consistent with Reference [50].

From Figure 3a–d and Figure 4, one can find that the influences of the structural parameters on absorption is independent of each other. Mode A and Mode B are generated by resonance of the internal structure and outer rings, respectively. It is indicated that our absorber offers great flexibility in the infrared regime. Therefore, by changing the dimensions of associated geometric parameters in the structure, two resonance wavelengths and the maximum absorption can be modulated individually while maintaining their dual-band characteristics.

**Figure 5:**
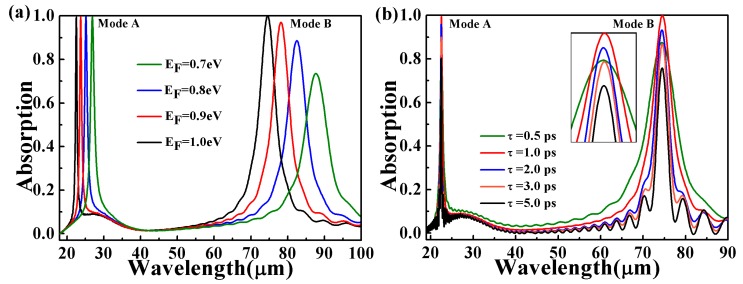
For TM polarization, (**a**) absorption spectrum of the absorber with different Fermi levels (the insert graph is absorption of Mode A) and (**b**) relaxation times. The insert graph is the absorption peak of Mode B.

However, for many metal absorbers or sensors, once their structures are fixed, their resonant wavelengths will no longer be tunable. Hence, we investigate the absorption spectrum of the absorber with the change of Fermi level (*E*_F_) and relaxation time (*τ*). Figure 5a shows the absorption spectrum of the absorber with different *E*_F_ values (0.7–1.0 eV). The wavelengths of the absorption peaks have an obvious blue shift as the Fermi level *E*_F_ increases, but Mode A moves slowly than Mode B. As a whole, high *E*_F_ has a higher absorption relative to low *E*_F_. The physical mechanism is related to the absorption of graphene increases with the increase of graphene Fermi level *E*_F_ and local electric field, and the enhancement of graphene SPR resonance.

Figure 5b shows the absorption spectrum for different *τ*, with *E*_F_ = 1.0 eV. The peak wavelength does not change, while the width of peak becomes narrow as *τ* increases. According to Equation (3), it can be known that the value of *τ* can be easily adjusted by changing μ (the carrier mobility). There is reference to provide methods for increasing carrier mobility by altering the surrounding environment, such as placing organic molecules on graphene [51]. More carrier mobility contributed to the plasmonic oscillation increases, resulting in higher absorption. But if *τ* is too large, the absorption will decrease due to most the energy being reflected. Therefore, as shown in Figure 5b, the maximum absorption increases at first and then decreases as *τ* increases. The absorption peaks can be saturated when *E*_F_ = 1.0 eV and *τ* = 1.0 ps. The above result implies that the absorption spectrum can be tuned by changing Fermi level and relaxation time without changing the absorber geometry.

**Figure 6:**
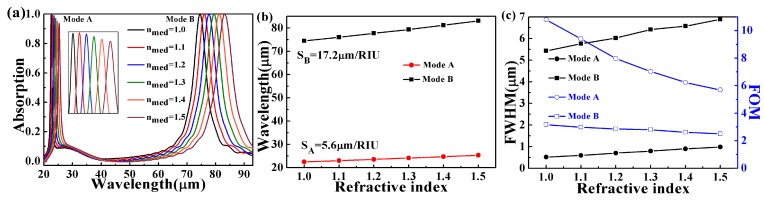
(**a**) Spectral shift of mode A and mode B with different sensing medium refractive indices (1.0–1.5) (the insert map is spectral shift of mode A); (**b**) a linear relationship of resonance wavelengths in response to changes in the refractive index; (**c**) FWHM (FWHM is the full width of half maximum) and figure of merit (FOM) of mode A and mode B in response to different sensing medium refractive indices.

To examine the sensing performance of the proposed dual-band PA, the dependence of the absorption spectrum on different surrounding refraction indices is calculated, with other parameters fixed (the same as in Figure 1). As shown in Figure 6a, the absorption peaks experience a red shift when the refractive index changes from 1.0 to 1.5. The sensitivity (S) and figure of merit (FOM) are the important parameters for sensors [52,53]. S and FOM can be expressed as S = Δλ/Δ*n* and FOM = S/FWHM, respectively [54,55]. In Figure 6b,c, the sensitivity can achieve 5.6 and 17.2 μm/RIU, and the FOM of around 10.8 and 3.2, respectively. For Mode A, it has narrow bandwidth and the FOM can be as high as 10.8. The dipolar resonance mode (Mode B) of the graphene metamaterials has the highest index sensitivity, while the quadrupolar mode (Mode A) displays the highest FOM due to the narrow bandwidth. Table 1 shows that the comparison of sensitivity (S) and the figure of merit (FOM) of different structures proposed in previous publications [44,56,57,58,59,60]. It can be seen clearly that the proposed structure has better sensing performance.

The above result implies that that the PA-based RI sensor is highly dependent on the refractive index and has a large sensing range. Therefore, the dual-band PA-based RI sensor can be conveniently used as a device for monitoring or sensing refractive index changes of a tested agent.

## 4. Conclusions

To conclude, we theoretically propose a dual-band plasmonic perfect absorber (PA) and refractive index (RI) sensor based on graphene metamaterials. The numerical results indicate that the absorber can achieve absorptions of 99.4% and 99.9% at 22.5 μm and 74.5 μm, respectively. Specifically, the absorption performance can be modulated by changing the Fermi level and the relaxation time without changing the geometric structure. Also, the PA-based RI sensor is highly dependent on the refractive index and has a large sensing range, which can achieve the sensitivities of around 5.6 and 17.2 μm/RIU, and FOM of around 10.8 and 3.2, respectively. We believe that our study could provide a potential application in biosensing, tunable spectral detecting, environmental monitoring and medical diagnostics.

## Figures and Tables

**Table 1:** Comparison of sensitivity (S) and the figure of merit (FOM) of different structures proposed in previous publications.

Reference	[44]	[56]	[57]	[58]	[59]	[60]	Proposed
Sensitivity (max) (μm/RIU)	15.006	9.59	11.5	0.43	0.64	1	17.2
FOM (max)	10.8	5.82	3.9	4.4	4.7	5	10.8
